# Multimodal Retinal Imaging of Intravascular Lipid in Severe/Extreme Hypertriglyceridemia

**DOI:** 10.1155/2023/6698239

**Published:** 2023-09-27

**Authors:** Christos Christakopoulos

**Affiliations:** Department of Ophthalmology, Zealand University Hospital, Næstved, Denmark

## Abstract

Retinal intravascular lipid aggregates were detected in the left eye in a patient with uncontrolled diabetes and very high blood triglycerides. The patient suffered visual loss in the left eye due to retinal ischemia. Optical coherence tomography, fluorescein angiography, infrared fundus photography, and autofluorescence studies of the retina demonstrated unique findings. Slowed choroidal and retinal flow was detected on fluorescein angiography, and a prominent middle layer membrane sign was present on OCT. Intravascular lipid reflectivity was manifest on retinal infrared and autofluorescence imaging. Eventually, insulin and statin therapy proved effective in reversing the vascular lesions, although retinal atrophy finally ensued. This report of a very rare clinical condition provides unique findings on multimodal retinal imaging and confirms the need for prompt insulin and statin therapy in severe/extreme hypertriglyceridemia and dysregulated diabetes. One similar case was reported in the past when multimodal imaging studies of the retina were not available. The lesions described herein should be differentiated from the more frequently encountered lipemia retinalis as they may confer worse prognosis.

## 1. Introduction

Hypertriglyceridemia occurs when blood triglyceride levels exceed 150 mg/dL. Severe/extreme hypertriglyceridemia should be considered when blood triglyceride levels exceed 1000 mg/dL [1]. In the past, white retinal intravascular lesions attributed to lipid aggregates were reported in one patient with diabetes and hypertriglyceridemia [2]. The case of a patient with known uncontrolled diabetes mellitus type 2 (DM2) and severe/extreme hypertriglyceridemia who presented with white retinal intravascular lesions is described herein.

## 2. Case Presentation

A 30-year-old man with DM2 presented to the Department of Neurology, Zealand University Hospital, with blurred vision on the left eye (LE).

Initial laboratory work-up during admission revealed very high hyperlipidemia with blood triglyceride levels of 2850 mg/dL (normal < 150 mg/dL), high blood cholesterol (6.9 mmol/L, normal < 5.0 mmol/L), blood glucose (23 mmol/L, normal: 4.2-6.3 mmol/L), and HbA1c (117 mmol/mol, normal < 48 mmol/mol) and normal haemoglobin (9.8 mmol/L), thrombocytes (156 × 10^9^/L), white blood cells (8.8 × 10^9^/L), and thyrotropin (1.9 × 10^−3^ IU/L). Computed tomography (CT) of the brain and transthoracic echocardiography were without abnormal findings, whereas ultrasound examination of the carotid arteries showed uniformly echogenic atherosclerotic plaque (type 4) at the level of carotid sinus on the left side and hypoechogenic plaque (type 2) on the right side, neither of them causing significant stenosis.

The patient admitted to have discontinued all medications for the last 18 months.

Because of persisting visual deterioration on the LE, he was referred to the Department of Ophthalmology, Zealand University Hospital.

**Figure 1:**
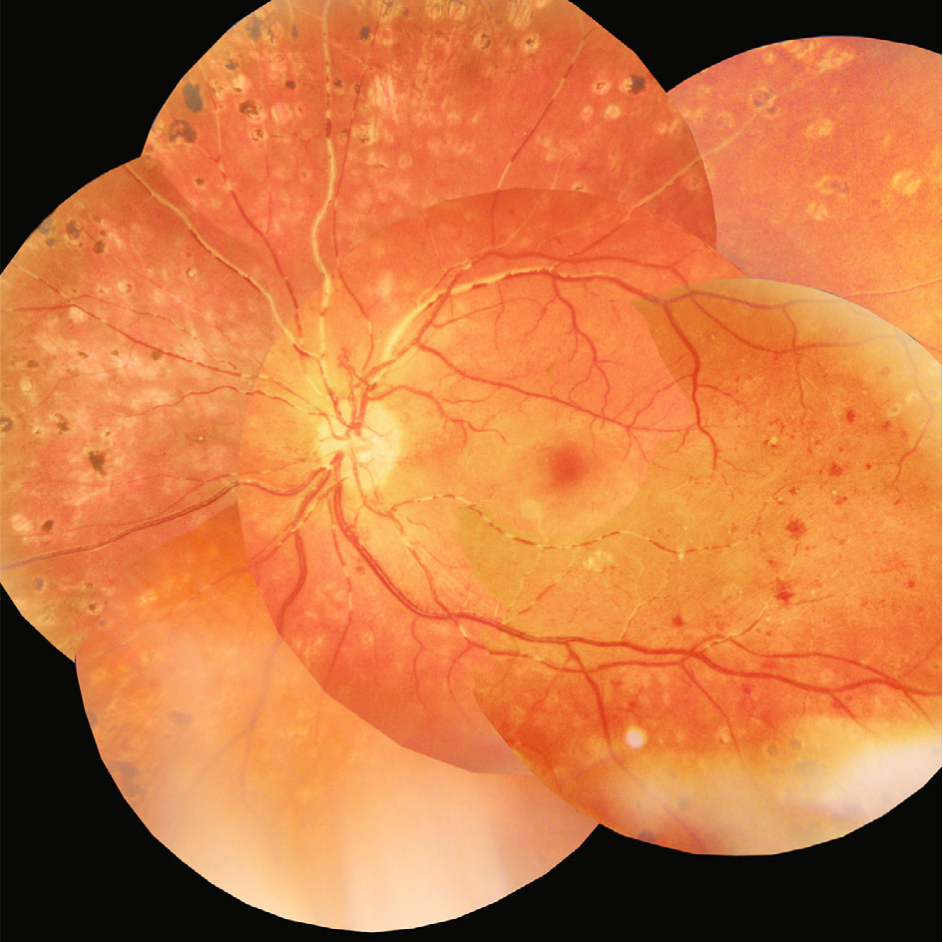
Composite fundus photograph depicting white vascular retinal lesions both in arteries and veins in the LE with a “cherry red spot” in macula.

On presentation, visual acuity on the right eye (RE) and LE was 20/20 and 20/60 (Snellen's acuity test). Slit lamp examination was normal in both eyes. Mydriatic fundus examination of the RE and LE showed proliferative diabetic retinopathy with new vessel formation on the optic disc and retina. Furthermore, fundus examination of the LE was notable for opaque milky white material segmentally filling the lumen of both arteries and veins. The white retinal aggregates were interrupted by blood filled vascular segments (Figure 1), and a “cherry red spot” was present in the macula (Figure 1).

**Figure 2:**
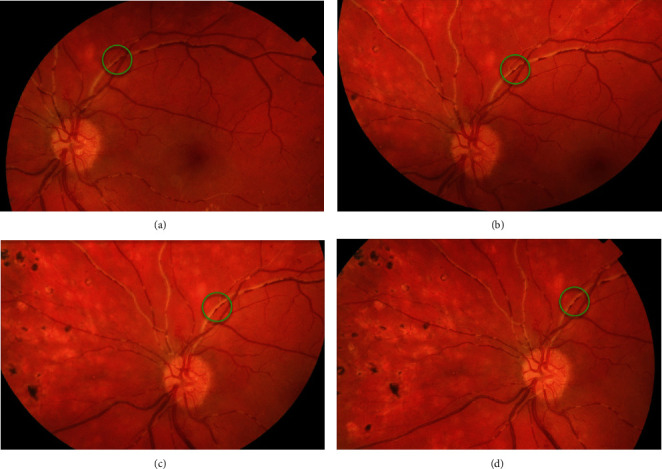
Serial fundus photography of the LE taken with intervals of 0 seconds (a), 21 seconds (b), 25 seconds (c), and 35 seconds (d) starting from (a) through (d). Red blood cellular aggregates can be traced moving downstream the superior temporal branch of central retinal vein (insert).

Serial fundus photography of the LE manifested movement of the intravascular lesions downstream the circulation (Figure 2).

**Figure 3:**
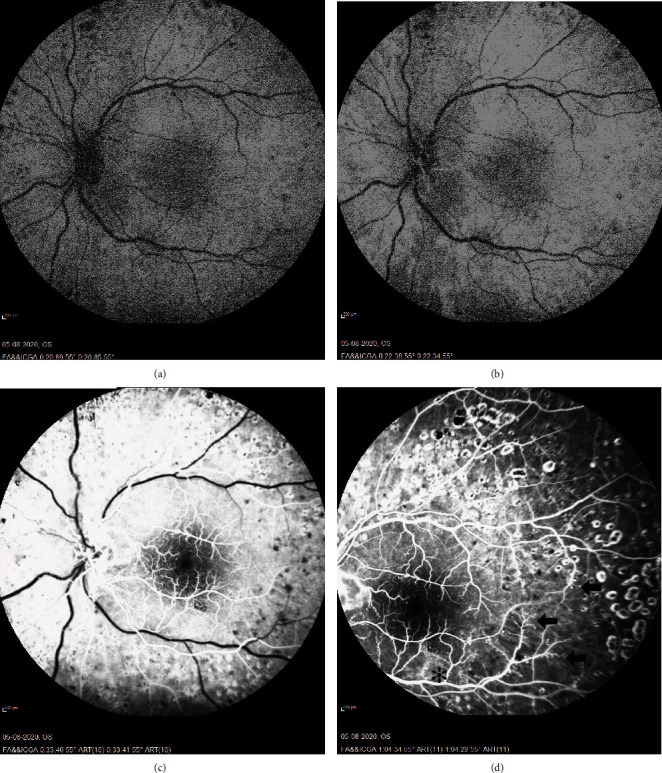
Fluorescein angiogram of the LE. Delayed choroidal filling at 20 seconds (a) followed by normal arterial phase 2 seconds later (b) and arteriovenous phase 11 seconds later (c). In the venous phase, 31 seconds later (d), arteriolar pruning with capillary dropout (arrowhead) and capillary bed leakage (asterisk) was noted. Microvascular pathology such as microaneurysms and intraretinal microvascular anomalies was ubiquitous.

Fluorescein angiography (FA) of the LE showed delayed choroidal and retinal arteriovenous phase that was protracted but without blockage in the flow of dye in the lipid laden vessels (Figure 3). The arteriovenous phase was delayed as well. In the venous phase, there was capillary bed leakage and focal areas of capillary dropout (Figure 3(d)).

**Figure 4:**
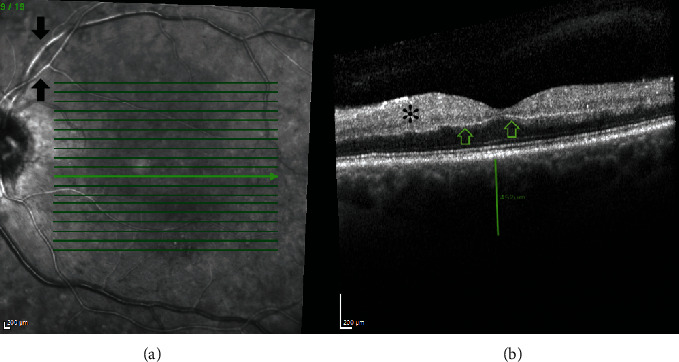
Near infrared image (a) shows hyperinfrared reflection from vascular aggregates in the affected vascular segments (arrowhead). Spectral domain OCT (Spectralis, Heidelberg Engineering™) (b) of the LE with a p-MLM (arrow) and hyperreflective inner retinal layers (asterisk).

On OCT (Spectralis, Heidelberg Engineering™), there was linear hyperdensity at the level of deep capillary plexus on the inner side of outer plexiform layer, compatible with a prominent middle limiting membrane sign (p-MLM) (Figure 4). There was noted increased thickness of the choroid on OCT (subfoveal choroidal thickness of 452 *μ*m) (Figure 4).

**Figure 5:**
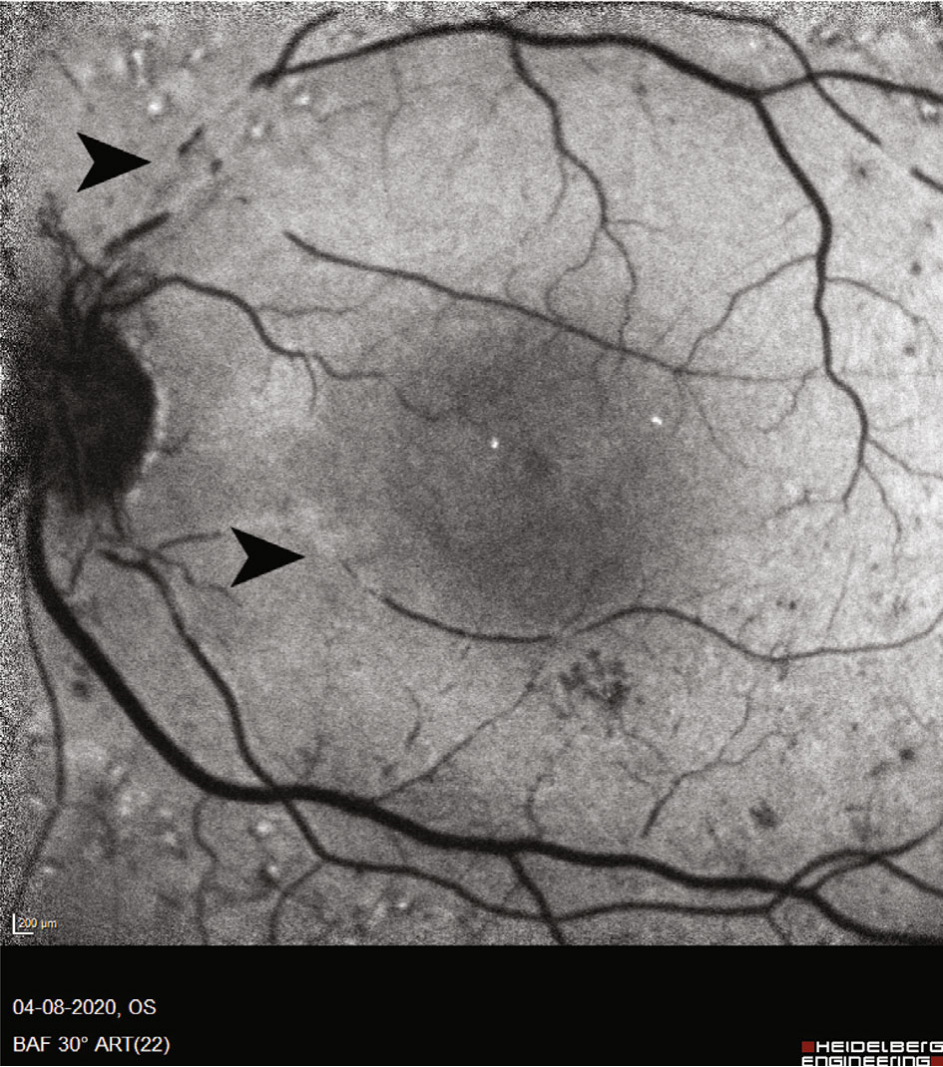
Fundus autofluorescence (Spectralis, Heidelberg Engineering™) of the LE, demonstrating increased autofluorescence at the observed white retinal vascular lesions (arrowhead).

Near infrared and autofluorescence imaging (Spectralis, Heidelberg Engineering™) of the fundus of the LE showed increased infrared reflection and autofluorescence from the affected arterial and venous segments (Figures 4 and 5).

**Figure 6:**
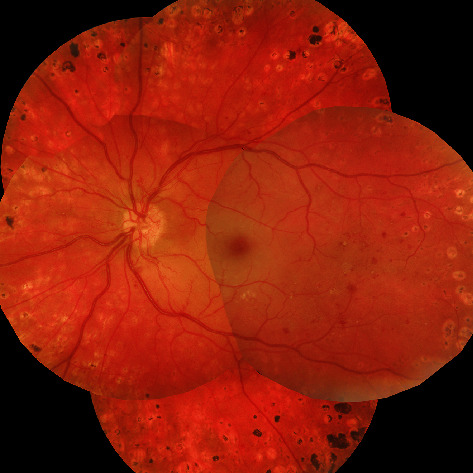
Composite fundus photograph of LE, one day after insulin and statin therapy initiation, showing resolution of the white vascular aggregates and persistence of the “cherry red spot” in macula.

**Figure 7:**
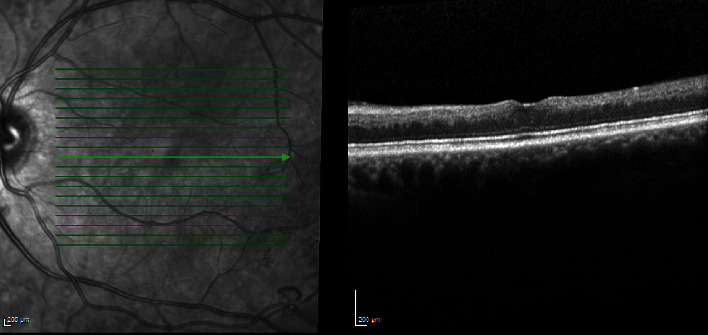
Spectral domain OCT (Spectralis, Heidelberg Engineering™) of the LE two years later, presenting atrophy of the inner retinal layers at the macular area.

The condition suggested retinal ischemia in LE in connection with the build-up of the opaque milky white fat aggregates in the lumen of retinal vessels. The patient was admitted to the department of Internal Medicine, Zealand University Hospital, and started on insulin and atorvastatin therapy with rapid elimination of the white lesions one day later (Figure 6). A decrease in blood trigylceride (394.7 mg/L), cholesterol (3.5 mmol/L) and glucose levels (4.0 mmol/L) were recorded the days following admission. Visual acuity at 5 weeks was 20/20 on the RE and 20/60 on the LE, and inner retinal layer thinning at the macula was evident (Figure 7).

Panretinal laser photocoagulation for proliferative diabetic retinopathy was subsequently performed in both eyes.

## 3. Discussion

The case described herein demonstrates milky white aggregates in the lumen of retinal vessels in the LE of a patient with poorly controlled DM2 and severe/extreme hypertriglyceridemia of 2850 mg/dL, followed by retinal ischemia and leading ultimately to retinal atrophy and visual deterioration. The lesions were similar to those reported by Kurz et al. [2], in a patient with DM and blood triglyceride levels of 2100 mg/dL with lipid filled retinal vessels and ischemic infarction of the adjacent retina.

In patients with DM, hypertriglyceridemia occurs due to increased plasma concentrations of VLDL with or without chylomicronemia [3].

The opaque white appearance of the vascular lesions and their prompt resolution after lipid lowering therapy suggest their origin in blood triglycerides. This is corroborated by the observed hyperautofluorescence of the involved retinal vessels and the increased infrared reflectivity on infrared fundus imaging [4, 5].

Rheological disturbances propagated by alterations in extraocular circulation on the left side in the setting of very high blood triglyceride levels may have caused the coalescence of intraluminal fat droplets into macroscopic fat macroglobules [6, 7].

Although the fluorescein angiogram did not show blockage at the involved vascular segments, increased latency of the retinal and choroidal circulation was evident in LE.

Moreover, the p-MLM sign indicates capillary perfusion insufficiency at the level of deep capillary plexus and is a harbinger of macular ischemia and atrophy [8] as documented on follow-up. On the other hand, the increased subfoveal choroidal thickness as documented by OCT could be caused by dilation of the choroidal vessels.

The above can be accounted for by a slower ocular blood flow due to hypertriglyceridemia [5].

Hypertriglyceridemia can cause increased plasma viscosity [9, 10] and is an independent risk factor for ischemic stroke [9, 11].

Increasing occupancy of the retinal vascular space by lipid globules leads to decreased hematocrit in the involved vessels [12] and an overall decreased hematocrit to viscosity ratio in the affected tissue [13].

*Τ*his condition should be distinguished from lipemia retinalis that occurs when blood triglyceride levels are in excess of 2000 mg/dL, with a uniform lactescent or creamy sheen of all retinal vessels and a salmon-coloured fundus in both eyes, not causing visual deterioration [14].

On the other hand, fibrinoplatelet emboli may fill large vascular segments but are limited to only the arterial side of circulation and have a dull gray appearance [15].

Reversal of the white lesions was observed one day after initiation of insulin and statin therapy. Insulin is particularly effective in diabetic hyperlipidemias as it induces the activity of lipoprotein lipase [16] and inhibits lipolysis from existing fat depots [17].

This case highlights the occurrence of a very rare phenotype in a patient with uncontrolled DM and severe/extreme hypertriglyceridemia that resulted in slowed retinal blood flow causing intrinsic retinal ischemia as shown by the recorded p-MLM sign. The recorded retinal imaging findings have not been reported before and are of clinical significance in the diagnostic work-up of intravascular white aggregates. The outcome of this case emphasizes the effectiveness of insulin and statin therapy in prompt resolution of the presumed lipid vascular aggregates and preservation of retinal function.

## Data Availability

This study is a one-patient case imaging study.
